# Exploring Impaired SERCA Pump-Caused Alternation Occurrence in Ischemia

**DOI:** 10.1155/2019/8237071

**Published:** 2019-11-12

**Authors:** Jiaqi Liu, Xiaoye Zhao, Yinglan Gong, Jucheng Zhang, Yunliang Zang, Ling Xia

**Affiliations:** ^1^Key Laboratory for Biomedical Engineering of Ministry of Education, Institute of Biomedical Engineering, Zhejiang University, Hangzhou 310027, China; ^2^Department of Medical Imaging Technology, North Minzu University, Yinchuan 750021, China; ^3^Connected Healthcare Big Data Research Center, Zhejiang Lab, Hangzhou 311100, China; ^4^Department of Clinical Engineering, 2nd Affiliated Hospital, School of Medicine, Zhejiang University, Hangzhou 310009, China; ^5^Computational Neuroscience Unit, Okinawa Institute of Science and Technology Graduate University, Okinawa 904-0495, Japan

## Abstract

Impaired sarcoplasmic reticulum (SR) calcium transport ATPase (SERCA) gives rise to Ca^2+^ alternans and changes of the Ca2+release amount. These changes in Ca^2+^ release amount can reveal the mechanism underlying how the interaction between Ca^2+^ release and Ca^2+^ uptake induces Ca^2+^ alternans. This study of alternans by calculating the values of Ca^2+^ release properties with impaired SERCA has not been explored before. Here, we induced Ca^2+^ alternans by using an impaired SERCA pump under ischemic conditions. The results showed that the recruitment and refractoriness of the Ca^2+^ release increased as Ca^2+^ alternans occurred. This indicates triggering Ca waves. As the propagation of Ca waves is linked to the occurrence of Ca^2+^ alternans, the “threshold” for Ca waves reflects the key factor in Ca^2+^ alternans development, and it is still controversial nowadays. We proposed the ratio between the diastolic network SR (NSR) Ca content (Ca_nsr_) and the cytoplasmic Ca content (Ca_*i*_) (Ca_nsr_/Ca_*i*_) as the “threshold” of Ca waves and Ca^2+^ alternans. Diastolic Ca_nsr_, Ca_*i*_, and their ratio were recorded at the onset of Ca^2+^ alternans. Compared with certain Ca_nsr_ and Ca_*i*_, the “threshold” of the ratio can better explain the comprehensive effects of the Ca^2+^ release and the Ca^2+^ uptake on Ca^2+^ alternans onset. In addition, these ratios are related with the function of SERCA pumps, which vary with different ischemic conditions. Thus, values of these ratios could be used to differentiate Ca^2+^ alternans from different ischemic cases. This agrees with some experimental results. Therefore, the certain value of diastolic Ca_nsr_/Ca_*i*_ can be the better “threshold” for Ca waves and Ca^2+^ alternans.

## 1. Introduction

Cardiac arrhythmia has long been associated with abnormal intracellular Ca^2+^ handling dynamics [1–4]. One useful diagnostic marker of arrhythmias is electrical alternans [5–8], which is expressed as alternated action potential durations (APDs) at the cellular level [9] and T waves on the electrocardiogram (ECG) [5], where the T wave stands for the repolarization of the ventricles and T wave alternans (TWA) indicates that the amplitude or the morphology of the T wave alternates beat-to-beat. The link between ischemia and alternans has been extensively explored [10]. Our previous simulations identified that hyperkalaemia, one component of ischemia, results in depolarization alternans [11]. Other two ischemic components, hypoxia and acidosis, lead to repolarization alternans by causing instabilities in calcium cycling [4, 11, 12]. Ca^2+^ alternans in ischemia can be taken as the arrhythmic triggers leading to afterdepolarization and also as the substrate facilitating reentry by inducing electrical alternans [4].

Alternans depends on instabilities of membrane voltage (*V*_m_) [13] or/and intracellular Ca^2+^ handling [1, 7, 9, 14–20], due to their bidirectional couplings [3, 9, 21]. For the latter, it is known that the Ca^2+^ handling includes Ca^2+^ influx and efflux [16], and its abnormality can arise from the dysfunction of sarcoplasmic reticulum (SR) calcium transport ATPase (SERCA) [11, 13, 14], ryanodine receptor (RyR2) [1, 4, 7, 14, 16], and Ca^2+^ leak [13]. Under ischemic conditions, both the Ca^2+^ release current (*I*_rel_) [4] and the Ca^2+^ uptake current (*I*_up_) decrease [4, 22] to facilitate the formation of Ca^2+^ alternans in an interactive manner. In this work, we will focus on Ca^2+^ alternans caused by an impaired SERCA pump in ischemia.

The sarcoplasmic reticulum (SR) Ca^2+^ release curve describes the nonlinear relationship between SR Ca^2+^ release content and diastolic SR Ca^2+^ content (Ca_sr_). The steep slope of this curve indicates that more Ca^2+^ is released at high diastolic SR Ca^2+^ content. In heart failure (HF), we have identified the primary role of the steep SR Ca^2+^ release curve in the genesis of alternans through simulation study [23]. Furthermore, the steep slope of the curve is also able to explain the impaired SERCA pump-caused Ca^2+^ alternans [19]. The onset of Ca^2+^ alternans in HF and in this study can be described as follows [9, 16]: when the slope of the curve is steep at certain Ca_sr_, a small increment of diastolic Ca_sr_ will result in a larger Ca^2+^ release, where released Ca^2+^ cannot be completely refilled back to the SR by impaired SERCA pumps. In the following heartbeat, the decreased Ca_sr_ gives rise to a smaller subsequent Ca^2+^ release. According to the above description, the steep slope of SR Ca^2+^ release curve provides the substrate for alternans onset and impaired SERCA pump enhances the susceptibility. Previous studies attribute the steep slope to Ca wave propagation [23–25] or the saturation of buffered Ca_sr_ [26]. In fact, the steep slope of the curve is directly linked to the change of *I*_rel_. Then, what are the detailed changes of *I*_rel_ to increase slopes? What is the factor that brings change to the *I*_rel_? To investigate these questions, we took use of “3R theory” [27] to find the answers. The “3R theory” defines three critical properties (α for “randomness”, *β* for “refractoriness,” and *γ* for “recruitment”) of a Ca spark, and we use the properties to analyze Ca^2+^ alternans. These properties are further introduced in the Materials and Methods section.

The propagation of Ca waves is linked to the onset of Ca^2+^ alternans [25, 27]. Although experimental and theoretical studies have investigated the development of Ca waves, there is a dispute regarding the definition of the “threshold” for Ca waves. Some experimental studies indicated Ca_sr_ as the “threshold” [24, 28, 29], while others highlighted the role of intracellular Ca^2+^ concentration (Ca_*i*_) [30–32]. We propose the ratio of diastolic network SR (NSR) Ca^2+^ content (Ca_nsr_) to diastolic Ca_*i*_ (Ca_nsr_/Ca_*i*_) as the “threshold” for Ca waves and Ca^2+^ alternans, which highlights both of their roles, and finally verify it by simulations. The “threshold” of diastolic Ca_nsr_/Ca_*i*_ is determined by thermodynamic constraints, which provides the theoretical basis for our new “threshold.” Moreover, this new “threshold” theory may help us better understand alternans and potentially provide a novel therapeutic strategy for alternans.

## 2. Materials and Methods

A thermodynamic model of SR Ca pump (SERCA pump model) [22] was integrated into the human epicardial (epi) ventricular cell model (O'Hara-Rudy dynamic (ORd) model) to simulate Ca^2+^ alternans [33]. The ORd model can reproduce the rate dependence of Ca^2+^ in experiments. The SERCA pump model is built based on experimental data of the rabbit and other animals. We used it to obtain the Ca^2+^ uptake rate per pump and multiplied a scale factor to calculate *I*_up_. The appropriate coefficient was determined by comparing the *I*_up_ amplitude produced by the original ORd epi cell model and the Ca^2+^ uptake rate per pump at steady state under normal conditions (this method was described in detail in our previous study [11]). The SERCA pump model incorporates the regulation effect of phospholamban (PLB) on Ca^2+^ uptake. Similar with the effect of increased pH (Figure 4 in [22]), PLB phosphorylation decreases the half-maximum Ca^2+^ uptake rate *K*_0.5_ [34] and increases SR Ca^2+^ uptake rate. During early phase of ischemia, the increased PLB phosphorylation helps to maintain the function of the SERCA pump [4]. After 20–30 minutes of ischemia, PLB dephosphorylation reduces SR Ca^2+^ uptake rate [4, 35]. In this work, we simulated the membrane voltage and ion concentrations after 10–20 minutes of ischemia, where phosphorylation level of PLB was kept the same as in control and the SERCA pump was impaired by ischemic components.

As shown below, two Ca^2+^ are translated from the cytoplasm to the SR during Ca^2+^ uptake [22]:

(1) $$\begin{array}{c} 2{\text{Ca}}_{\text{i}}^{2+}+\text{MgATP}+{\text{H}}_{2}\text{O}⇌2{\text{Ca}}_{\text{sr}}^{2+}+\text{MgADP}+\text{Pi}+{\text{H}}^{+} \end{array}$$

To investigate the effect of the ischemia-impaired SERCA pump on *I*_rel_ at stable state, we first compared calcium transients between control and ischemia and then applied the “3R theory” to analyze the changes of *I*_rel_ achieved by decreased *I*_up_. This investigation explained how *I*_up_ cooperated with *I*_rel_ to cause Ca^2+^ alternans.

### 2.1. Conditions Setting in the Simulations

Ischemic conditions contribute to compromised metabolism and thus lead to decreased function of SERCA pumps. Specifically, hypoxia decreases intracellular ATP concentration ([ATP]_*i*_) and increases intracellular ADP concentration ([ADP]_*i*_) [36]. Meanwhile, inorganic phosphate (Pi) in the cytoplasm increases [37] and pH is decreased by acidosis [12]. According to these experimental data, we simulated three cases of ischemia (Table 1) with the cycle length (CL) of 250 ms and 350 ms, respectively to obtain Ca^2+^ alternans. After 1000 beats, action potentials (APs) and Ca transients were taken to be stable. Then, we analyzed them in the subsequent 1000 beats.

The CL also affects whether Ca^2+^ alternans can occur or not in different ischemic conditions. We attempted to find the ranges of CL in which Ca^2+^ alternans could arise in these three cases. In our simulations, the starting CL is 250 ms and the increasing step is 10 ms. Finally, we determined the ranges of CL in ischemic cases 1, 2, and 3, which are from 250 ms to 280 ms, from 250 ms to 380 ms, and from 250 ms to 300 ms, respectively.

### 2.2. The SR Ca^2+^ Release Curve

In our simulations, the total amount of diastolic SR Ca^2+^ (Ca_sr_total_ (mmol)) comprised the amount of diastolic NSR Ca^2+^ and the junctional SR (JSR) Ca^2+^. The amount of Ca^2+^ release (Ca_release(*k*)_ (mmol)) was expressed as the integral of the Ca^2+^ release flux (*J*_rel(*k*)_ (mmol/L/ms)) on the *k*^th^ beat (equation (4)). Then, we used the ratio of Ca_release(*k*)_ to Ca_sr_total(*k*−1)_ to represent the fraction of SR Ca^2+^ release:

(2) $$\begin{array}{c} {\text{Ca}}_{\text{sr}\_\text{total}\left(k\right)}=v_{\text{nsr}}*{\text{Ca}}_{\text{nsr}\left(k\right)}+v_{\text{jsr}}*\left({\text{Ca}}_{\text{jsr}\_\text{free}\left(k\right)}+{\text{Ca}}_{\text{jsr}\_\text{buff}\left(k\right)}\right), \end{array}$$

(3) $$\begin{array}{c} {\text{Ca}}_{\text{sr}\left(k\right)}=\frac{{\text{Ca}}_{\text{sr}\_\text{total}\left(k\right)}}{v_{\text{nsr}}+v_{\text{jsr}}}, \end{array}$$

(4) $$\begin{array}{c} {\text{Ca}}_{\text{release}\left(k\right)}=v_{\text{jsr}}\int_{0}^{T}J_{\text{rel}\left(k\right)}\text{d}t\text{,} \end{array}$$

where JSR Ca^2+^ included free and buffered Ca^2+^ (Ca_jsr_free_ (mmol/L) and Ca_jsr_buff_ (mmol/L)); *v*_nsr_ and *v*_jsr_ represented the volume of NSR and JSR; and Ca_sr(*k*)_ (mmol/L) and Ca_nsr(*k*)_ (mmol/L), respectively, referred to the diastolic SR and NSR Ca^2+^ content on the *k*^th^ beat.

### 2.3. Calculating Values of *α*, *β*, and *γ* according to “3R” Theory

In the spatially distributed calcium cycling model developed by Rovetti et al. [27], SR Ca^2+^ is released through CRUs. One CRU is set to have six neighbors in the 3D-distribution cell simulation [27]. As shown in equations (5) and (6), *N*_0_ represents the total number of CRUs and *N*_*K*_ is the number of that activated on the *k*^th^ beat [27], where *α* represents the probability of a Ca spark being activated spontaneously or by the L-type Ca^2+^ current (*I*_CaL_); *β* is the probability of a Ca spark triggered on the *k*^th^ beat being unavailable during the (*k* + 1)^th^ beat; *γ* indicates the probability of a Ca spark recruiting one of its neighboring; and *f* represents the percentage of secondary Ca sparks in the remaining available CRUs [27]. The number of CRUs activated on the (*k* + 1)^th^ beat is given as follows [27]:

(5) $$\begin{array}{c} N_{K+1}=\left(N_{0}–\beta *N_{K}\right)*\left(\alpha +1-\alpha *f\right), \end{array}$$

(6) $$\begin{array}{c} f=1-{\left(1-\alpha *\gamma *\left(1-\beta *\frac{N_{K}}{N_{0}}\right)\right)}^{6}, \end{array}$$

(7) $$\begin{array}{c} \langle \Delta \text{Ca}\rangle =\left({\text{Ca}}_{\text{SR}}-\langle {\text{Ca}}_{\text{b}}\rangle \right)*\frac{N_{K}}{N_{0}}, \end{array}$$

where <ΔCa> is the average SR Ca^2+^ depletion of each CRU and Ca_sr_ is the average Ca^2+^ content of each CRU before release [27]. <Ca_b_> refers to the average Ca^2+^ content of these *N*_*k*_ CRUs after they sparked [27]. We put Ca_release(*k*)_ and Ca_sr_total_ to replace <ΔCa> and Ca_SR_ to obtain equation (8). The left-hand side is SR Ca^2+^ concentration depletion. Thus, Ca_release(*k*)_ and Ca_sr_total(*k*−1)_, calculated from our simulations (equations (2) and (4)), were linked with *N*_*K*_ and *N*_0_.

(8) $$\begin{array}{c} \frac{{\text{Ca}}_{\text{release}\left(k\right)}}{v_{\text{nsr}}+v_{\text{jsr}}}=\left(\frac{{\text{Ca}}_{\text{sr\_total}\left(k-1\right)}}{v_{\text{nsr}}+v_{\text{jsr}}}-\frac{\langle {\text{Ca}}_{\text{b}}\rangle }{v_{\text{nsr}}+v_{\text{jsr}}}\right)*\frac{N_{K}}{N_{0}}. \end{array}$$

*N*_*K*_ and *N*_0_ in equation (8) were replaced by Ca_release(*k*)_, Ca_sr_total(*k*−1)_, and <Ca_b_>. Thus, the relationship between properties of RyRs and our simulation results was built.

(9) $$\begin{array}{c} \frac{{\text{Ca}}_{\text{release}\left(k+1\right)}}{{\text{Ca}}_{{\text{sr}}_{\text{total}\left(k\right)}}-\langle {\text{Ca}}_{\text{b}}\rangle }=\left(1-\beta *\frac{{\text{Ca}}_{\text{release}\left(k\right)}}{{\text{Ca}}_{{\text{sr}}_{\text{total}\left(k-1\right)}}-\langle {\text{Ca}}_{\text{b}}\rangle }\right)*\left(\alpha +\left(1-\alpha \right)*\left(1-{\left(1-\alpha *\gamma *\left(1-\beta *\frac{{\text{Ca}}_{\text{release}\left(k\right)}}{{\text{Ca}}_{\text{sr\_total}\left(k-1\right)}-\langle {\text{Ca}}_{\text{b}}\rangle }\right)\right)}^{6}\right)\right), \end{array}$$

where *α*, *β*, *γ*, and <Ca_b_> are unknown parameters and others could be obtained from our simulation results. To obtain these unknown parameters, we solved equation (9) by using the MATLAB built-in lsqcurvefit function. First, the inputs of Ca_release(*k*)_ and Ca_sr_total(*k*−1)_ were calculated from simulations. Meanwhile, initial *α*, *β*, and *γ* were set as random values from zero to one and the initial <Ca_b_>/(*v*_nsr_ + *v*_jsr_) was from zero to the maximum Ca_sr_total_/(*v*_nsr_ + *v*_jsr_). Then, these values were input to solve equation (9). Specifically, when we calculated these parameters during the short period of alternans formation, the groups of inputs were too few to obtain accurate values of these unknown parameters. We solved <Ca_b_> in equation (9) before and after alternans onset in advance and take the value of it as a constant to input equation (9). Thus, the number of unknowns is decreased, and the remaining three unknowns are able to be obtained during the short period of alternans formation.

### 2.4. Definition of the Occurrence of Ca^2+^ Alternans

Ca^2+^ alternans was supposed to occur when the following criteria were met:

(10) $$\begin{array}{c} \frac{\left({\text{Ca}}_{\text{amplitude}\left(k+1\right)}-{\text{Ca}}_{\text{amplitude}\left(k\right)}\right)}{{\text{Ca}}_{\text{amplitude}\left(k\right)}}\geq 5\%, \end{array}$$

where Ca_amplitude(*k*)_ is defined as the amplitude of Ca^2+^ transient on the *k*^th^ beat.

## 3. Results

**Figure 1:**
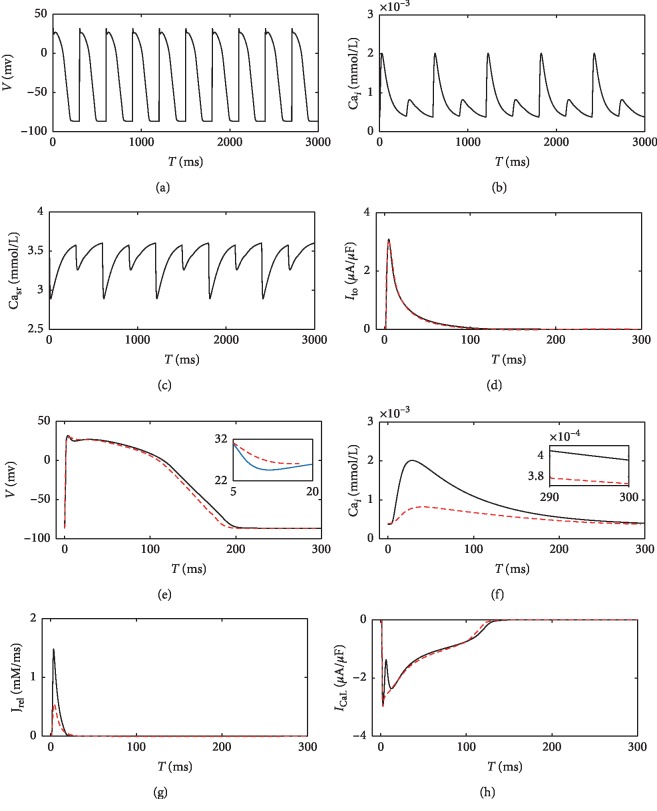
Alternans of APs and Ca transients and the relevant currents in ischemic case 2. Alternated APs, Ca_*i*_, and Ca_sr_ are shown in (a)–(c), respectively. Aligned *I*_to_, APs, Ca_*i*_, *J*_rel_, and *I*_CaL_ between two continuous APs are compared in (d)–(h), respectively, where the solid black lines stand for ischemic conditions and the dashed red lines stand for control condition. CL is 300 ms.

According to equation (1), increased [ADP]_*i*_, [Pi]_*i*_, and [H^+^]_*i*_ and decreased [ATP]_*i*_ result in less Ca^2+^ transported from the cytoplasm to the SR. The function of the SERCA pump is impaired by ischemia. As shown in Figure 1, the amplitudes of Ca_*i*_ and Ca_sr_ alternate obviously. In contrast, diastolic Ca_*i*_ alternates slightly (inset of Figure 1(e)). APs also show slight alternans (Figure 1(d)), due to the Ca^2+^ alternans-caused fluctuation of *I*_CaL_. A larger Ca^2+^ release decreases *I*_CaL_, makes the transient outward current (*I*_to_) more prominent, and leads to a slightly deeper notch of the AP. Subsequently, the voltage-dependent repolarization currents cause different repolarization phases.

**Figure 2:**
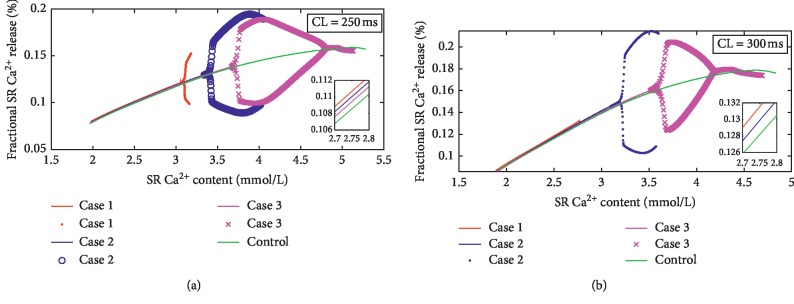
SR Ca^2+^ release curves under control and ischemic conditions (cases 1, 2, and 3). (a) CL = 250 ms. (b) CL = 300 ms.

As shown in Figure 2, Ca^2+^ alternans can be observed in all three ischemic cases when CL = 250 ms. However, when the CL increases to 300 ms, it can only be observed in cases 2 and 3. In case 1, the maximum Ca_sr_ with CL of 300 ms does not reach the value of Ca_sr_ at which bifurcations occur with CL of 250 ms. In addition, the slopes of curves change slightly before alternans onset (inset of Figure 2), but the values of Ca_sr_ change obviously when bifurcations occur. The values of Ca_sr_ at which bifurcations occur decrease with the ischemic degree at the same CLs.

**Figure 3:**
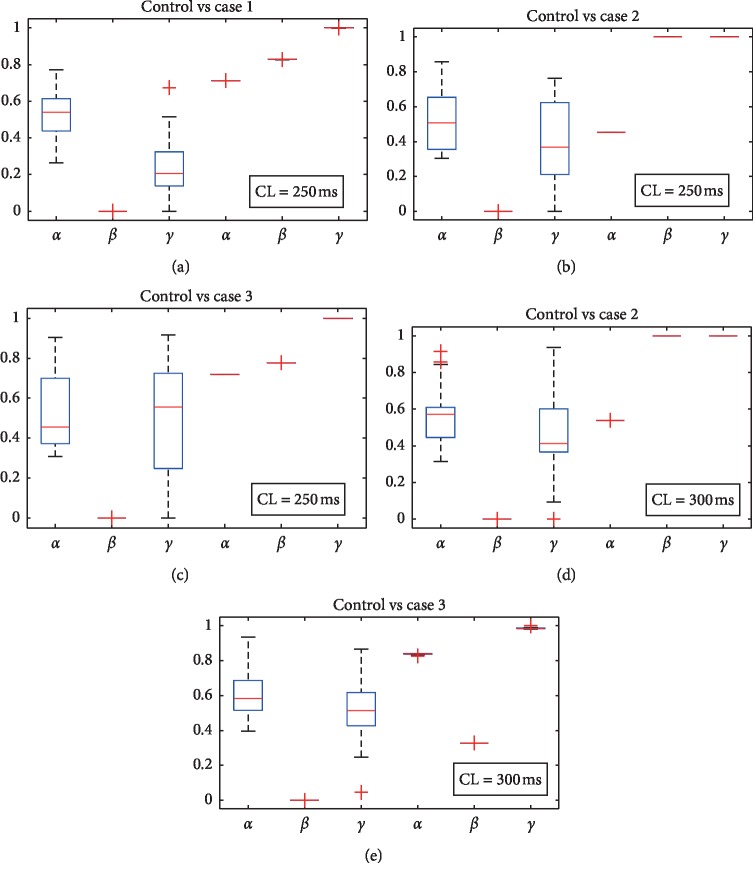
Values of *α*, *β*, and *γ* during the formation of Ca^2+^ alternans in control and ischemic cases. Twenty groups of parameter values are included in each box plot. In each panel, the left three box plots are in control conditions and the right three are under ischemia. The ranges of Ca_sr_ are the same in every two contrasting groups. (a) Ca_sr_ ranges from 3 to 3.7 mmol/L with the CL of 300 ms. (b) Ca_sr_ ranges from 3.2 to 3.9 mmol/L with the CL of 300 ms. (c) Ca_sr_ ranges from 2.8 to 3.15 mmol/L and the CL is 250 ms. (d) Ca_sr_ ranges from 3 to 4 mmol/L and the CL is 250 ms. (e) Ca_sr_ ranges from 3.5 to 4.4 mmol/L and the CL is 250 ms.

In Figure 3, *α*, *β*, and *γ* were obtained during the formation of Ca^2+^ alternans under different ischemic conditions. Compared with the control group, *β* and *γ* increase obviously in all ischemic conditions. In control condition, average *β* and *γ* are 0 and 0.42, respectively. They both increase to 1 in ischemic case 2 with CL of 300 ms. Nonetheless, *α* does not vary a lot.

**Figure 4:**
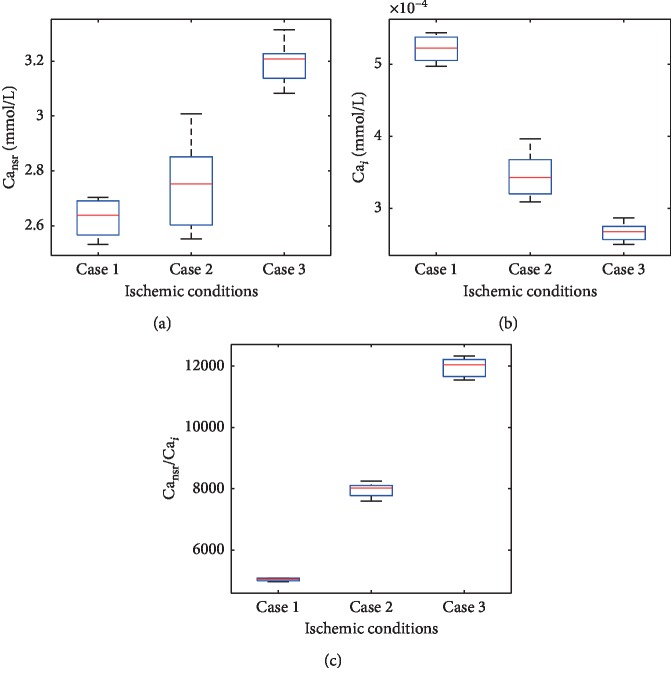
Diastolic Ca_nsr_, Ca_*i*_, and Ca_nsr_/Ca_*i*_ at the onset of Ca^2+^ alternans. Ca^2+^ alternans arises at different ranges of CLs in three ischemic cases. The ranges of CLs in ischemic cases 1, 2, and 3 are from 250 ms to 280 ms, from 250 ms to 380 ms, and from 250 ms to 300 ms.

**Figure 5:**
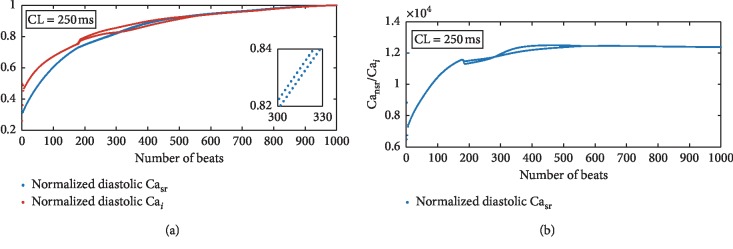
Normalized diastolic Ca_*i*_ (dashed red line in (a)), Ca_sr_ (dashed blue line in (a)), and diastolic Ca_nsr_/Ca_*i*_ (b) under transient Ca^2+^ alternans. These values are recorded under ischemic case 3 during the 1000 consecutive beats.

The values of diastolic Ca_nsr_, Ca_*i*_, and Ca_nsr_/Ca_*i*_ in Figure 4 were recorded once Ca^2+^ alternans occurred in ischemia. On the one hand, Ca^2+^ alternans in case 1 or case 2 cannot be distinguished by the recorded values of Ca_nsr_ (Figure 4(a)). On the other hand, there is a small difference between the values of the recorded Ca_*i*_ in case 2 and those in case 3 (Figure 4(b)). In contrast, the ratios vary obviously with ischemic cases. Compared with the effect of CLs, the degree of ischemia (values of [ADP]_*i*_, [Pi]_*i*_, [H^+^]_*i*_, and [ATP]_*i*_) affects the ratios more effectively. Furthermore, how diastolic Ca_sr_, Ca_*i*_, and their ratio change with sequent heartbeats is analyzed under transient Ca^2+^ alternans (Figure 5). Ca^2+^ alternans lasts for some beats and gradually disappears in ischemic case 3 when CL = 250 ms. In the whole process of Ca^2+^ alternans development, diastolic Ca_nsr_, Ca_*i*_, and their ratio fluctuate and increase (Figure 5). After Ca^2+^ alternans disappears, the ratio remains a constant value (Figure 5(b)) while the other two continue to increase (Figure 5(a)), where Ca_sr_ and Ca_*i*_ are divided by their maximum values, respectively, to get normalized values, which are no bigger than one. This will facilitate the comparison in Figure 5(a).

## 4. Discussion

Consistent with previous study [25], fluctuations of Ca_sr_ are observed during the impaired SERCA pump-caused Ca^2+^ alternans (Figures 1(c) and 5(a)). However, the slight fluctuations of Ca_sr_ alone are insufficient to maintain Ca^2+^ alternans without the steep slope of Ca^2+^ release curve. The large fraction of Ca^2+^ release is demonstrated to generate Ca^2+^ alternans [16, 23, 25, 39]. Figure 2 shows that the curve slopes change slightly before the onset of alternans in different ischemic conditions. Subsequently, the obvious bifurcations occur in the curve. These obvious changes are the dominant factors to cause alternans. To elucidate how these bifurcations happen, we analyze how *I*_rel_ is affected by impaired SERCA pump in the period of bifurcations occurrence.

*I*_rel_ can be regarded as a collective effect of Ca sparks. During the formation of alternans, changes in properties of Ca sparks reflect how *I*_rel_ is affected by the impaired SERCA pump. The values of *β* and *γ* increase obviously in ischemic groups compared to control without Ca^2+^ alternans (Figure 3). Rovetti et al. [27] concluded that large *β* and *γ* together with properly chosen *α* promote alternans. Our results confirmed their prediction (Figure 3).

Large *β* indicates long refractory period of RyRs when the CL is unchanged [27], implying a long time required for complete recovery of RyRs. Our results show that large *β* can be induced by the impaired SERCA pump. This can be easily understood through introducing a Ca^2+^ cycling hypothesis [16]: cytosolic Ca^2+^, taken up by the SERCA pump in the NSR, is released by the RyRs channels in the JSR. Thus, the process of transporting Ca^2+^ from the NSR to the JSR results in a delayed Ca^2+^ release after the uptake. The impaired SERCA pump slows the Ca^2+^ recycling process and increases RyRs refractory period. During the slow Ca^2+^ recycling process, the amount of Ca^2+^ reaching the release sites fluctuates, leading to alternated large and small Ca^2+^ releases. This hypothesis presents a possible mechanism underlying how SERCA pump modulates the refractoriness of the RyRs.

According to “3R theory,” large *γ* indicates frequent spark-induced sparks, which probably produce Ca waves. The propagation of Ca waves requires Ca diffusion to take effect. Ca diffusion is also shown to influence another behavior of synchronizing local Ca^2+^ release [40]. The degree of synchronization increases obviously when the “threshold” CL for alternans is approaching [40]. Because of the different dependence of pacing, Ca waves differ with the synchronization. However, when the synchronization occurs, Ca diffusion is also more likely to propagate Ca waves. The occurrence of Ca^2+^ alternans also promotes Ca^2+^ wave to propagate. On the other hand, the prolonged refractory period can produce alternated Ca waves by regulating the numbers of available CRUs. In all, large *γ* can either directly result from Ca^2+^ alternans or be induced by the prolonged refractory period.

Although we have investigated how *I*_rel_ changes during the formation of Ca^2+^ alternans, the timing for these changes taking place is still unknown. Large *γ* is associated with the propagation of Ca waves. Ca waves are also linked to the onset of Ca^2+^ alternans. Figure 2 shows that Ca^2+^ alternans begin at some value of Ca_sr_. That is, that value of Ca_sr_ is able to be regarded as the “threshold” for Ca waves [24, 28, 29]. This idea is also supported by the fact that increased Ca_sr_ increases *α* and initiates Ca waves [24, 28]. However, if Ca_sr_ is believed to be the “threshold” for Ca waves and Ca^2+^ alternans, then Ca^2+^ alternans should not disappear as Ca_sr_ keeps rising (Figure 5(a)). In fact, whether the “threshold” of Ca_sr_ determines Ca waves onset or not is also debated in other studies that tried to link Ca_*i*_ to the occurrence of Ca waves [30–32]. Moreover, although previous experimental study [28] supports the idea that the “threshold” of Ca_sr_ determines Ca waves onset, diastolic Ca_*i*_ has also been associated with the frequency of release (Figures 2(a) and 2(b) of [28]). Undoubtedly, diastolic Ca_*i*_ also exerts influence in producing Ca waves and Ca^2+^ alternans. Therefore, we propose diastolic Ca_nsr_/Ca_*i*_ as the “threshold” of Ca waves and Ca^2+^ alternans, which reflects the roles of both Ca_sr_ and Ca_*i*_ in the formation of Ca waves and Ca^2+^ alternans.

SERCA pumps contribute to maintain the Ca^2+^ concentration gradient between the SR and the cytoplasm. Since the Ca^2+^ uptake sites are in the NSR, diastolic Ca_nsr_/Ca_*i*_ is related to the Ca^2+^ uptake. According to equation (1), the maximum uptake rate is modulated by ischemic conditions ([ADP]_*i*_, [ATP]_*i*_, [Pi]_*i*_, and [H^+^]_*i*_). This means the maximum diastolic Ca_nsr_/Ca_*i*_ can be affected by ischemic cases. In addition, the onset of alternans is induced by ischemia, and thus its “threshold” is taken to be different with ischemic degrees. In Figure 4, the “threshold” of diastolic Ca_nsr_/Ca_*i*_ differs with ischemic conditions. As a contrast, neither Ca_*i*_ nor Ca_nsr_ is able to distinguish different ischemic cases. In Figure 5, before the onset of Ca^2+^ alternans, Ca_sr_ and Ca_*i*_ increase. Correspondingly, *I*_up_ goes on retaking the increasing released Ca^2+^ and diastolic Ca_nsr_/Ca_*i*_ keeps rising. However, to what extent *I*_up_ and diastolic Ca_nsr_/Ca_*i*_ can increase is limited by the thermodynamic constrains. Ca^2+^ alternans and Ca waves form when the unbalance between the Ca^2+^ uptake and Ca^2+^ release occurs. Subsequently, as diastolic Ca_*i*_ and Ca_nsr_ keep on increasing, the Ca^2+^ uptake rate increases enough to uptake all released Ca^2+^ and Ca^2+^ alternans disappears. This final constant value of diastolic Ca_nsr_/Ca_*i*_ indicates the new balance between the Ca^2+^ release and Ca^2+^ uptake. On the other hand, if diastolic Ca_*i*_ or Ca_nsr_ is the “threshold” for Ca waves and Ca^2+^ alternans, these two increasing values will initiate larger Ca waves and Ca^2+^ alternans will not disappear.

Xie et al. [15] demonstrated the SR Ca^2+^ efflux cooperates with the influx to affect the “threshold” for alternans. An intermediate SR Ca^2+^ uptake rate and a larger SR Ca^2+^ release work synergistically to produce alternans at longer CLs [16]. According to our new “threshold” theory, the limited increase of *I*_up_ contributes to unbalanced *I*_up_ and *I*_rel_ and promotes abnormal intracellular Ca^2+^ handling. In addition, other studies demonstrated that the properties of RyRs affect the “threshold” of Ca_sr_ for alternans [24, 41, 42]. When the open probability of RyRs channels increases at the same Ca_sr_ [41], the Ca^2+^ uptake rate becomes larger accordingly. As the physiological conditions ([ADP]_*i*_, [ATP]_*i*_, [Pi]_*i*_, and [H^+^]_*i*_) are identical, the maximum Ca^2+^ uptake rate is kept the same. Thus, maximum *I*_up_ occurs at a lower Ca_sr_, resulting in a reduced “threshold” of Ca_sr_ for alternans (Figure 3 of [24] and Figure 4 of [41]).

This “threshold” theory provides a new idea for changes of *I*_rel_ during the formation of Ca^2+^ alternans. Our new “theory” shows that the unbalance between *I*_up_ and *I*_rel_ begins at the “threshold” of diastolic Ca_nsr_/Ca_*i*_. These two currents interact with each other and result in Ca^2+^ alternans. We should also note that Ca^2+^ alternans of case 3 is transient (Figure 5), which suggests that when *I*_up_ and *I*_rel_ get balanced, Ca^2+^ alternans is suppressed. Therefore, this “threshold” theory may disclose a novel therapeutic strategy for Ca^2+^ alternans. In theory, keeping the ratio below the “threshold” stops Ca^2+^ alternans occurrence. In clinical, the treatment aiming at the interaction between these two currents may have a promising effect. Although direct therapeutic tools modulating the SR release channels have not been fully developed [2], the new proposed “threshold” theory can be regarded as a strong guideline for searching for new therapeutic targets.

## 5. Conclusion

The integrated cell model can be used to simulate the SERCA pump function in specific ischemic conditions, whereas the original ORd model cannot be used. The simulated results indicate that Ca waves can be induced by impaired SERCA pump and thus give rise to Ca^2+^ alternans. That is, these components of Ca cycling interact with each other to affect Ca^2+^ alternans development. In addition, compared with isolated changes of diastolic Ca_sr_ and Ca_*i*_, the value of diastolic Ca_nsr_/Ca_*i*_ is more appropriate to function as the “threshold” for alternans. By defining this new “threshold,” we can better explain how the interplay between the *I*_up_ and *I*_rel_ causes alternans. Furthermore, this proposed “threshold” theory may help find therapeutic targets for suppressing Ca^2+^ alternans.

## 6. Limitations

This model is just used to simulate the impaired SERCA pump function during ischemia, whereas L-type calcium and other currents are also affected under ischemia, and these changes are not included in our model. We need to further improve this integrated model to simulate more accurate ischemic conditions. It is noted that the values of the three properties can be different by using different groups of inputs. The range of Ca_*i*_ chosen in our simulation can also determine the results by influencing the inputs. We also need to take use of other cell models to calculate values of these properties during Ca^2+^ alternans occurrence and identify our “threshold” theory. In addition, although we conclude that *I*_up_ and *I*_rel_ interact to produce Ca^2+^ alternans, we just identify how the Ca release is affected by impaired SERCA pump, and the effect of *I*_rel_ on *I*_up_ should also be identified in detail in the future.

## Figures and Tables

**Table 1:** Parameters setting under different conditions.

Conditions	[ATP]_*i*_ (mmol/L)	pH
Ischemic case 1	3	6.5
Ischemic case 2	3.5	6.6
Ischemic case 3	4	6.7
Control condition	9.8	7

The values of [ATP]_*i*_ and pH were in the ranges during early phase of ischemia [12, 38]. In ischemia, [ADP]_*i*_ and [Pi]_*i*_ were set to 0.2 mmol/L [36] and 30 mmol/L [37], respectively. In control, the two were set to 0.015 mmol/L and 0 mmol/L, respectively.

## Data Availability

The data used to build graphs in this study are available from the corresponding author upon request.

## Supplementary Materials

Supplementary Materials Our supplementary files include the modified human ventricular cell model, codes, and data for plotting figures in our papers.
