# Supplementary material for: Exploring Impaired SERCA Pump-Caused Alternation Occurrence in Ischemia
Source: Comput Math Methods Med. 2019 Nov 12;2019:8237071. doi: 10.1155/2019/8237071 (PMC6885202; doi:10.1155/2019/8237071)
Supplement: Supplementary Materials — Our supplementary files include the modified human ventricular cell model, codes, and data for plotting figures in our papers. [file 8237071.f1.zip › 8237071.f1/pdf format/supplementary material_codes.pdf]

```
%%cardiac cell model

%%%%%%%%%%%%%%%%%%%%%%%%%%%%%%%%%%%%
%%%%% Cardiac cell model %%%%%%
%%%%%%%%%%%%%%%%%%%%%%%%%%%%%%%%%%%%

function output=model(t,X,flag_ode)

CL = 300;%ms

celltype=1; %endo = 0, epi = 1, M = 2

%extracellular ionic concentrations
nao=140.0;
cao=1.8;
ko=5.4;

%physical constants
R=8314.0;
T=310.0;
F=96485.0;

%cell geometry
L=0.01;
rad=0.0011;
vcell=1000*3.14*rad*rad*L;
Ageo=2*3.14*rad*rad+2*3.14*rad*L;
Acap=2*Ageo;
vmyo=0.68*vcell;
vnsr=0.0552*vcell;
vjsr=0.0048*vcell;
vss=0.02*vcell;

%parameters setting in control (used in SERCA three-state model)
   ADP = 0.015;
   Pi = 0;
   ATP_in=9.8;
   Hi=10^(-4);

%give names to the state vector values
v=X(1);
nai=X(2);
nass=X(3);
ki=X(4);
```

```
kss=X(5);
cai=X(6);
cass=X(7);
cansr=X(8);
cajsr=X(9);
m=X(10);
hf=X(11);
hs=X(12);
j=X(13);
hsp=X(14);
jp=X(15);
mL=X(16);
hL=X(17);
hLp=X(18);
a=X(19);
iF=X(20);
iS=X(21);
ap=X(22);
iFp=X(23);
iSp=X(24);
d=X(25);
ff=X(26);
fs=X(27);
fcaf=X(28);
fcas=X(29);
jca=X(30);
nca=X(31);
ffp=X(32);
fcafp=X(33);
xrf=X(34);
xrs=X(35);
xs1=X(36);
xs2=X(37);
xk1=X(38);
Jrelnp=X(39);
Jrelp=X(40);
CaMKt=X(41);

%%%%%%%%%%%%%%%%%%%%%%%%%%%%%%%%%%%%%%%%%%%%%%%%%%%%%%%%%%%%%%%%%%%%%%%%

%CaMK constants
KmCaMK=0.15;

aCaMK=0.05;
```

```
bCaMK=0.00068;
CaMKo=0.05;
KmCaM=0.0015;
%update CaMK
CaMKb=CaMKo*(1.0-CaMKt)/(1.0+KmCaM/cass);
CaMKa=CaMKb+CaMKt;
dCaMKt=aCaMK*CaMKb*(CaMKb+CaMKt)-bCaMK*CaMKt;

%%%%%%%%%%%%%%%%%%%%%%%%%%%%%%%%%%%%%%%%%%%%%%%%%%%%%%%%%%%%%%%%%%%%%%%%%

%reversal potentials
ENa=(R*T/F)*log(nao/nai);
EK=(R*T/F)*log(ko/ki);
PKNa=0.01833;
EKs=(R*T/F)*log((ko+PKNa*nao)/(ki+PKNa*nai));

%convenient shorthand calculations
vffrt=v*F*F/(R*T);
vfrt=v*F/(R*T);

%%%%%%%%%%%%%%%%%%%%%%%%%%%%%%%%%%%%%%%%%%%%%%%%%%%%%%%%%%%%%%%%%%%%%%%%%

%calculate INa
mss=1.0/(1.0+exp((-(v+39.57))/9.871));
tm=1.0/(6.765*exp((v+11.64)/34.77)+8.552*exp(-(v+77.42)/5.955));
dm=(mss-m)/tm;
hss=1.0/(1+exp((v+82.90)/6.086));
thf=1.0/(1.432e-5*exp(-(v+1.196)/6.285)+6.149*exp((v+0.5096)/20.27));
ths=1.0/(0.009794*exp(-(v+17.95)/28.05)+0.3343*exp((v+5.730)/56.66));
Ahf=0.99;
Ahs=1.0-Ahf;
dhf=(hss-hf)/thf;
dhs=(hss-hs)/ths;
h=Ahf*hf+Ahs*hs;
jss=hss;
tj=2.038+1.0/(0.02136*exp(-(v+100.6)/8.281)+0.3052*exp((v+0.9941)/38.45));
dj=(jss-j)/tj;
hssp=1.0/(1+exp((v+89.1)/6.086));
thsp=3.0*ths;
dhsp=(hssp-hsp)/thsp;
hp=Ahf*hf+Ahs*hsp;
tjp=1.46*tj;
djp=(jss-jp)/tjp;
GNa=75;
```

```
fINap=(1.0/(1.0+KmCaMK/CaMKa));
INa=GNa*(v-ENa)*m^3.0*((1.0-fINap)*h*j+fINap*hp*jp);

%calculate INaL
mLss=1.0/(1.0+exp((-(v+42.85))/5.264));
tmL=tm;
dmL=(mLss-mL)/tmL;
hLss=1.0/(1.0+exp((v+87.61)/7.488));
thL=200.0;
dhL=(hLss-hL)/thL;
hLssp=1.0/(1.0+exp((v+93.81)/7.488));
thLp=3.0*thL;
dhLp=(hLssp-hLp)/thLp;
GNaL=0.0075;
if celltype==1
    GNaL=GNaL*0.6;
end
fINaLp=(1.0/(1.0+KmCaMK/CaMKa));
INaL=GNaL*(v-ENa)*mL*((1.0-fINaLp)*hL+fINaLp*hLp);

%calculate Ito
ass=1.0/(1.0+exp((-(v-14.34))/14.82));
ta=1.0515/(1.0/(1.2089*(1.0+exp(-(v-18.4099)/29.3814)))+3.5/(1.0+exp((v+100.0)/29.3814)));
da=(ass-a)/ta;
iss=1.0/(1.0+exp((v+43.94)/5.711));
if celltype==1
    delta_epi=1.0-(0.95/(1.0+exp((v+70.0)/5.0)));
else
    delta_epi=1.0;
end
tiF=4.562+1/(0.3933*exp((-(v+100.0))/100.0)+0.08004*exp((v+50.0)/16.59));
tiS=23.62+1/(0.001416*exp((-(v+96.52))/59.05)+1.780e-8*exp((v+114.1)/8.079));
tiF=tiF*delta_epi;
tiS=tiS*delta_epi;
AiF=1.0/(1.0+exp((v-213.6)/151.2));
AiS=1.0-AiF;
diF=(iss-iF)/tiF;
diS=(iss-iS)/tiS;
i=AiF*iF+AiS*iS;
assp=1.0/(1.0+exp((-(v-24.34))/14.82));
dap=(assp-ap)/ta;
dti_develop=1.354+1.0e-4/(exp((v-167.4)/15.89)+exp(-(v-12.23)/0.2154));
dti_recover=1.0-0.5/(1.0+exp((v+70.0)/20.0));
tiFp=dti_develop*dti_recover*tiF;
```

```
tiSp=dti_develop*dti_recover*tiS;
diFp=(iss-iFp)/tiFp;
diSp=(iss-iSp)/tiSp;
ip=AiF*iFp+AiS*iSp;
Gto=0.02;
if celltype==1
        Gto=Gto*4.0;
elseif celltype==2
        Gto=Gto*4.0;
end
fItop=(1.0/(1.0+KmCaMK/CaMKa));
Ito=Gto*(v-EK)*((1.0-fItop)*a*i+fItop*ap*ip);


[vcycle, pp] =    Tran_SERCA_3state(cai,cajsr,Hi,ATP_in,ADP,Pi); %adding the SERCA three-state modelin the cell model


dss=1.0/(1.0+exp((-(v+3.940))/4.230));
td=0.6+1.0/(exp(-0.05*(v+6.0))+exp(0.09*(v+14.0)));
dd=(dss-d)/td;
fss=1.0/(1.0+exp((v+19.58)/3.696));
tff=7.0+1.0/(0.0045*exp(-(v+20.0)/10.0)+0.0045*exp((v+20.0)/10.0));
tfs=1000.0+1.0/(0.000035*exp(-(v+5.0)/4.0)+0.000035*exp((v+5.0)/6.0));
Aff=0.6;
Afs=1.0-Aff;
dff=(fss-ff)/tff;
dfs=(fss-fs)/tfs;
f=Aff*ff+Afs*fs;
fcass=fss;
tfcaf=7.0+1.0/(0.04*exp(-(v-4.0)/7.0)+0.04*exp((v-4.0)/7.0));
tfcas=100.0+1.0/(0.00012*exp(-v/3.0)+0.00012*exp(v/7.0));
Afcaf=0.3+0.6/(1.0+exp((v-10.0)/10.0));
Afcas=1.0-Afcaf;
dfcaf=(fcass-fcaf)/tfcaf;
dfcas=(fcass-fcas)/tfcas;
fca=Afcaf*fcaf+Afcas*fcas;
tjca=75.0;
djca=(fcass-jca)/tjca;
tffp=2.5*tff;
dffp=(fss-ffp)/tffp;
fp=Aff*ffp+Afs*fs;
tfcafp=2.5*tfcaf;
dfcafp=(fcass-fcafp)/tfcafp;
```

```
fcap=Afcaf*fcafp+Afcas*fcas;
Kmn=0.002;
k2n=1000.0;
km2n=jca*1.0;
anca=1.0/(k2n/km2n+(1.0+Kmn/cass)^4.0);
dnca=anca*k2n-nca*km2n;
PhiCaL=4.0*vffrt*(cass*exp(2.0*vfrt)-0.341*cao)/(exp(2.0*vfrt)-1.0);
PhiCaNa=1.0*vffrt*(0.75*nass*exp(1.0*vfrt)-0.75*nao)/(exp(1.0*vfrt)-1.0);
PhiCaK=1.0*vffrt*(0.75*kss*exp(1.0*vfrt)-0.75*ko)/(exp(1.0*vfrt)-1.0);
zca=2.0;
PCa=0.0001;
if celltype==1
    PCa=PCa*1.2;
elseif celltype==2
    PCa=PCa*2.5;
end
PCap=1.1*PCa;
PCaNa=0.00125*PCa;
PCaK=3.574e-4*PCa;
PCaNap=0.00125*PCap;
PCaKp=3.574e-4*PCap;
fICaLp=(1.0/(1.0+KmCaMK/CaMKa));
ICaL=(1.0-fICaLp)*PCa*PhiCaL*d*(f*(1.0-nca)+jca*fca*nca)+fICaLp*PCap*PhiCaL*d*(fp*(1.0-
nca)+jca*fcap*nca);
ICaNa=(1.0-fICaLp)*PCaNa*PhiCaNa*d*(f*(1.0-
nca)+jca*fca*nca)+fICaLp*PCaNap*PhiCaNa*d*(fp*(1.0-nca)+jca*fcap*nca);
ICaK=(1.0-fICaLp)*PCaK*PhiCaK*d*(f*(1.0-
nca)+jca*fca*nca)+fICaLp*PCaKp*PhiCaK*d*(fp*(1.0-nca)+jca*fcap*nca);

%calculate IKr
xrss=1.0/(1.0+exp((-(v+8.337))/6.789));
txrf=12.98+1.0/(0.3652*exp((v-31.66)/3.869)+4.123e-5*exp((-(v-47.78))/20.38));
txrs=1.865+1.0/(0.06629*exp((v-34.70)/7.355)+1.128e-5*exp((-(v-29.74))/25.94));
Axrf=1.0/(1.0+exp((v+54.81)/38.21));
Axrs=1.0-Axrf;
dxrf=(xrss-xrf)/txrf;
dxrs=(xrss-xrs)/txrs;
xr=Axrf*xrf+Axrs*xrs;
rkr=1.0/(1.0+exp((v+55.0)/75.0))*1.0/(1.0+exp((v-10.0)/30.0));
GKr=0.046;
if celltype==1
    GKr=GKr*1.3;
elseif celltype==2
    GKr=GKr*0.8;
```

```
end
IKr=GKr*sqrt(ko/5.4)*xr*rkr*(v-EK);

%calculate IKs
xs1ss=1.0/(1.0+exp((-(v+11.60))/8.932));
txs1=817.3+1.0/(2.326e-4*exp((v+48.28)/17.80)+0.001292*exp((-(v+210.0))/230.0));
dxs1=(xs1ss-xs1)/txs1;
xs2ss=xs1ss;
txs2=1.0/(0.01*exp((v-50.0)/20.0)+0.0193*exp((-(v+66.54))/31.0));
dxs2=(xs2ss-xs2)/txs2;
KsCa=1.0+0.6/(1.0+(3.8e-5/cai)^1.4);
GKs=0.0034;
if celltype==1
    GKs=GKs*1.4;
end
IKs=GKs*KsCa*xs1*xs2*(v-EKs);

xk1ss=1.0/(1.0+exp(-(v+2.5538*ko+144.59)/(1.5692*ko+3.8115)));
txk1=122.2/(exp((-(v+127.2))/20.36)+exp((v+236.8)/69.33));
dxk1=(xk1ss-xk1)/txk1;
rk1=1.0/(1.0+exp((v+105.8-2.6*ko)/9.493));
GK1=0.1908;
if celltype==1
    GK1=GK1*1.2;
elseif celltype==2
    GK1=GK1*1.3;
end
IK1=GK1*sqrt(ko)*rk1*xk1*(v-EK);

%calculate INaCa_i
kna1=15.0;
kna2=5.0;
kna3=88.12;
kasymm=12.5;
wna=6.0e4;
wca=6.0e4;
wnaca=5.0e3;
kcaon=1.5e6;
kcaoff=5.0e3;
qna=0.5224;
qca=0.1670;
hca=exp((qca*v*F)/(R*T));
hna=exp((qna*v*F)/(R*T));
h1=1+nai/kna3*(1+hna);
```

```
h2=(nai*hna)/(kna3*h1);
h3=1.0/h1;
h4=1.0+nai/kna1*(1+nai/kna2);
h5=nai*nai/(h4*kna1*kna2);
h6=1.0/h4;
h7=1.0+nao/kna3*(1.0+1.0/hna);
h8=nao/(kna3*hna*h7);
h9=1.0/h7;
h10=kasymm+1.0+nao/kna1*(1.0+nao/kna2);
h11=nao*nao/(h10*kna1*kna2);
h12=1.0/h10;
k1=h12*cao*kcaon;
k2=kcaoff;
k3p=h9*wca;
k3pp=h8*wnaca;
k3=k3p+k3pp;
k4p=h3*wca/hca;
k4pp=h2*wnaca;
k4=k4p+k4pp;
k5=kcaoff;
k6=h6*cai*kcaon;
k7=h5*h2*wna;
k8=h8*h11*wna;
x1=k2*k4*(k7+k6)+k5*k7*(k2+k3);
x2=k1*k7*(k4+k5)+k4*k6*(k1+k8);
x3=k1*k3*(k7+k6)+k8*k6*(k2+k3);
x4=k2*k8*(k4+k5)+k3*k5*(k1+k8);
E1=x1/(x1+x2+x3+x4);
E2=x2/(x1+x2+x3+x4);
E3=x3/(x1+x2+x3+x4);
E4=x4/(x1+x2+x3+x4);
KmCaAct=150.0e-6;
allo=1.0/(1.0+(KmCaAct/cai)^2.0);
zna=1.0;
JncxNa=3.0*(E4*k7-E1*k8)+E3*k4pp-E2*k3pp;
JncxCa=E2*k2-E1*k1;
Gncx=0.0008;
if celltype==1
    Gncx=Gncx*1.1;
elseif celltype==2
    Gncx=Gncx*1.4;
end
INaCa_i=0.8*Gncx*allo*(zna*JncxNa+zca*JncxCa);
```

```
%calculate INaCa_ss
h1=1+nass/kna3*(1+hna);
h2=(nass*hna)/(kna3*h1);
h3=1.0/h1;
h4=1.0+nass/kna1*(1+nass/kna2);
h5=nass*nass/(h4*kna1*kna2);
h6=1.0/h4;
h7=1.0+nao/kna3*(1.0+1.0/hna);
h8=nao/(kna3*hna*h7);
h9=1.0/h7;
h10=kasymm+1.0+nao/kna1*(1+nao/kna2);
h11=nao*nao/(h10*kna1*kna2);
h12=1.0/h10;
k1=h12*cao*kcaon;
k2=kcaoff;
k3p=h9*wca;
k3pp=h8*wnaca;
k3=k3p+k3pp;
k4p=h3*wca/hca;
k4pp=h2*wnaca;
k4=k4p+k4pp;
k5=kcaoff;
k6=h6*cass*kcaon;
k7=h5*h2*wna;
k8=h8*h11*wna;
x1=k2*k4*(k7+k6)+k5*k7*(k2+k3);
x2=k1*k7*(k4+k5)+k4*k6*(k1+k8);
x3=k1*k3*(k7+k6)+k8*k6*(k2+k3);
x4=k2*k8*(k4+k5)+k3*k5*(k1+k8);
E1=x1/(x1+x2+x3+x4);
E2=x2/(x1+x2+x3+x4);
E3=x3/(x1+x2+x3+x4);
E4=x4/(x1+x2+x3+x4);
KmCaAct=150.0e-6;
allo=1.0/(1.0+(KmCaAct/cass)^2.0);
JncxNa=3.0*(E4*k7-E1*k8)+E3*k4pp-E2*k3pp;
JncxCa=E2*k2-E1*k1;
INaCa_ss=0.2*Gncx*allo*(zna*JncxNa+zca*JncxCa);

%calculate INaK
k1p=949.5;
k1m=182.4;
k2p=687.2;
k2m=39.4;
```

```
k3p=1899.0;
k3m=79300.0;
k4p=639.0;
k4m=40.0;
Knai0=9.073;
Knao0=27.78;
delta=-0.1550;
Knai=Knai0*exp((delta*v*F)/(3.0*R*T));
Knao=Knao0*exp(((1.0-delta)*v*F)/(3.0*R*T));
Kki=0.5;
Kko=0.3582;
MgADP=0.05;
MgATP=9.8;
Kmgatp=1.698e-7;
H=1.0e-7;
eP=4.2;
Khp=1.698e-7;
Knap=224.0;
Kxkur=292.0;
P=eP/(1.0+H/Khp+nai/Knap+ki/Kxkur);
a1=(k1p*(nai/Knai)^3.0)/((1.0+nai/Knai)^3.0+(1.0+ki/Kki)^2.0-1.0);
b1=k1m*MgADP;
a2=k2p;
b2=(k2m*(nao/Knao)^3.0)/((1.0+nao/Knao)^3.0+(1.0+ko/Kko)^2.0-1.0);
a3=(k3p*(ko/Kko)^2.0)/((1.0+nao/Knao)^3.0+(1.0+ko/Kko)^2.0-1.0);
b3=(k3m*P*H)/(1.0+MgATP/Kmgatp);
a4=(k4p*MgATP/Kmgatp)/(1.0+MgATP/Kmgatp);
b4=(k4m*(ki/Kki)^2.0)/((1.0+nai/Knai)^3.0+(1.0+ki/Kki)^2.0-1.0);
x1=a4*a1*a2+b2*b4*b3+a2*b4*b3+b3*a1*a2;
x2=b2*b1*b4+a1*a2*a3+a3*b1*b4+a2*a3*b4;
x3=a2*a3*a4+b3*b2*b1+b2*b1*a4+a3*a4*b1;
x4=b4*b3*b2+a3*a4*a1+b2*a4*a1+b3*b2*a1;
E1=x1/(x1+x2+x3+x4);
E2=x2/(x1+x2+x3+x4);
E3=x3/(x1+x2+x3+x4);
E4=x4/(x1+x2+x3+x4);
zk=1.0;
JnakNa=3.0*(E1*a3-E2*b3);
JnakK=2.0*(E4*b1-E3*a1);
Pnak=30;
if celltype==1
    Pnak=Pnak*0.9;
elseif celltype==2
    Pnak=Pnak*0.7;
```

```
end
INaK=Pnak*(zna*JnakNa+zk*JnakK);

%calculate IKb
xkb=1.0/(1.0+exp(-(v-14.48)/18.34));
GKb=0.003;
if celltype==1
        GKb=GKb*0.6;
end
IKb=GKb*xkb*(v-EK);

%calculate INab
PNab=3.75e-10;
INab=PNab*vffrt*(nai*exp(vfrt)-nao)/(exp(vfrt)-1.0);

%calculate ICab
PCab=2.5e-8;
ICab=PCab*4.0*vffrt*(cai*exp(2.0*vfrt)-0.341*cao)/(exp(2.0*vfrt)-1.0);

%calculate IpCa
GpCa=0.0005;
IpCa=GpCa*cai/(0.0005+cai);

%%%%%%%%%%%%%%%%%%%%%%%%%%%%%%%%%%%%%%%%%%%%%%%%%%%%%%%%%%%%%%%%%%%%

%calculate the stimulus current, Istim
amp=-80.0;
duration=0.5;

if (t-floor(t/CL)*CL <= duration)    && (t-floor(t/CL)*CL >0)
        Istim=amp;
else
        Istim=0;
end

%udtate the membrane voltage
dv=-
(INa+INaL+Ito+ICaL+ICaNa+ICaK+IKr+IKs+IK1+INaCa_i+INaCa_ss+INaK+INab+IKb+IpCa+
ICab+Istim);


%calculate diffusion fluxes
JdiffNa=(nass-nai)/2.0;
JdiffK=(kss-ki)/2.0;
```

```
Jdiff=(cass-cai)/0.2;

%calculate ryanodione receptor calcium induced calcium release from the jsr
bt=4.75;
a_rel=0.5*bt;
Jrel_inf=a_rel*(-ICaL)/(1.0+(1.5/cajsr)^8.0);
if celltype==2
    Jrel_inf=Jrel_inf*1.7;
end
tau_rel=2*bt/(1.0+0.0123/cajsr);

if tau_rel<0.001
   tau_rel=0.001;
end
dJrelnp=(Jrel_inf-Jrelnp)/tau_rel;
btp=1.25*bt;
a_relp=0.5*btp;
Jrel_infp=a_relp*(-ICaL)/(1.0+(1.5/cajsr)^8.0);
if celltype==2
    Jrel_infp=Jrel_infp*1.7;
end
tau_relp=2*btp/(1.0+0.0123/cajsr);
if tau_relp<0.001
   tau_relp=0.001;
end

dJrelp=(Jrel_infp-Jrelp)/tau_relp;
fJrelp=(1.0/(1.0+KmCaMK/CaMKa));
Jrel=(1.0-fJrelp)*Jrelnp+fJrelp*Jrelp;

%calculate serca pump, ca uptake flux
Jupnp=0.004375*cai/(cai+0.00092);
Jupp=2.75*0.004375*cai/(cai+0.00092-0.00017);
if celltype==1
    Jupnp=Jupnp*1.3;
    Jupp=Jupp*1.3;
end
fJupp=(1.0/(1.0+KmCaMK/CaMKa));
% Jup=(1.0-fJupp)*Jupnp+fJupp*Jupp-Jleak; %%%original equation in Ord model
Jleak=0.0039375*cansr/15.0;
Jup = 0.00179*vcycle-Jleak; %%% calculate Jup by using the cycle rate after adding the SERCA three-state model
%calculate tranlocation flux
Jtr=(cansr-cajsr)/100.0;
```

```
%calcium buffer constants
cmdnmax=0.05;
if celltype==1
    cmdnmax=cmdnmax*1.3;
end
kmcmdn=0.00238;
trpnmax=0.07;
kmtrpn=0.0005;
BSRmax=0.047;
KmBSR=0.00087;
BSLmax=1.124;
KmBSL=0.0087;
csqnmax=10.0;
kmcsqn=0.8;

%update intracellular concentrations, using buffers for cai, cass, cajsr
dnai=-(INa+INaL+3.0*INaCa_i+3.0*INaK+INab)*Acap/(F*vmyo)+JdiffNa*vss/vmyo;
dnass=-(ICaNa+3.0*INaCa_ss)*Acap/(F*vss)-JdiffNa;

dki=-(Ito+IKr+IKs+IK1+IKb+Istim-2.0*INaK)*Acap/(F*vmyo)+JdiffK*vss/vmyo;
dkss=-(ICaK)*Acap/(F*vss)-JdiffK;

Bcai=1.0/(1.0+cmdnmax*kmcmdn/(kmcmdn+cai)^2.0+trpnmax*kmtrpn/(kmtrpn+cai)^2.0);
dcai=Bcai*(-(IpCa+ICab-2.0*INaCa_i)*Acap/(2.0*F*vmyo)-Jup*vnsr/vmyo+Jdiff*vss/vmyo);

Bcass=1.0/(1.0+BSRmax*KmBSR/(KmBSR+cass)^2.0+BSLmax*KmBSL/(KmBSL+cass)^2.0);
dcass=Bcass*(-(ICaL-2.0*INaCa_ss)*Acap/(2.0*F*vss)+Jrel*vjsr/vss-Jdiff);

dcansr=Jup-Jtr*vjsr/vnsr;

Bcajsr=1.0/(1.0+csqnmax*kmcsqn/(kmcsqn+cajsr)^2.0);
dcajsr=Bcajsr*(Jtr-Jrel);

cajsr_buff = csqnmax*(cajsr/(cajsr+kmcsqn));

%%%%%%%%%%%%%%%%%%%%%%%%%%%%%%%%%%%%%%%%%%%%%%%%%%%%%%%%%%%%%%%%%%%%%%%%%%%%%%%%
output the state venctor when ode_flag==1, and the calculated currents and fluxes otherwise
if flag_ode==1
   output=[dv dnai dnass dki dkss dcai dcass dcansr dcajsr dm dhf dhs dj dhsp djp dmL dhL
dhLp da diF diS dap diFp diSp dd dff dfs dfcaf dfcas djca dnca dffp dfcafp dxrf dxrs dxs1 dxs2
dxk1 dJrelnp dJrelp dCaMKt]';

else
```

```
    output=[INa INaL Ito ICaL IKr IKs IK1 INaCa_i INaCa_ss INaK   IKb INab ICab IpCa Jdiff JdiffNa JdiffK Jup Jleak Jtr Jrel CaMKa Istim cajsr_buff]';
end
```

%SERCA THREE-STATE MODEL

```
%%%%%%%%%%%%%%%%%%%%%%%%%%%%%%%%%%%
%%%%% Three-state SERCA model %%%%%
%%%%%%%%%%%%%%%%%%%%%%%%%%%%%%%%%%%

% This function outputs the SERCA three-state model cycle rate
% and phosphorylation proportion as a function of Cai, Casr, ATP, ADP and Pi
% All concentrations are in units of mM - including H_i

% Author: Kenneth Tran
% Last updated: 25/09/08
function [v_cycle, phosph] = Tran_SERCA_3state(Ca_i,Ca_sr,H_i,ATP,ADP,Pi)
	%Assigning the parameter values
	k_p1 = 25900;
	k_p2 = 2540;
	k_p3 = 20.5;
	k_m1 = 2;
	k_m2 = 67200;
	k_m3 = 149;
	kdcai = 0.91;
	kdcasr = 2.24;
	kdh1 = 1.09e-5;
	kdhi = 3.54e-3;
	kdhsr = 1.05e-8;
	kdh = 7.24e-5;
	n_H = 2.0;
	T_Cai = Ca_i/kdcai;
	T_Casr = Ca_sr/kdcasr;
	T_H1 = H_i/kdh1;
	T_Hi = (H_i^n_H)/kdhi;
	T_Hsr = (H_i.^n_H)/kdhsr;
	T_H = H_i/kdh;

	% Apparent forward rate constants
	a_p1 = k_p1*ATP;
	a_p2 = k_p2*T_Cai*T_Cai/(T_Cai^2 + T_Cai^2.*T_Hi + T_Hi*(1+T_H1));
	a_p3 = (k_p3*T_Hsr)/(T_Casr*T_Casr*T_H + T_H*(1) + T_Hsr*(1+T_H));

	% Apparent backward rate constants
	a_m1 = k_m1*T_Hi/(T_Cai^2 + T_Cai^2.*T_Hi + T_Hi*(1+T_H1));
	a_m2 = (k_m2*ADP*T_Casr*T_Casr*T_H)/(T_Casr*T_Casr*T_H + T_H + T_Hsr*(1+T_H));
	a_m3 = k_m3*Pi;
```

```
% Calculating steady state flux using the diagram method
s1 = a_p2*a_p3 + a_m1*a_p3 + a_m1*a_m2;
s2 = a_p1*a_p3 + a_m2*a_p1 + a_m2*a_m3;
s3 = a_p1*a_p2 + a_m3*a_m1 + a_m3*a_p2;

sum = s1 + s2 + s3;

% Calculating the cycle rate
v_cycle = (a_p1*a_p2*a_p3 - a_m1*a_m2*a_m3)/sum;

% Proportion of states that are phosphorylated
phosph = (s3)/(s1+s2+s3);
```

```
%SOLVE FUNCTION

%%%%%%%%%%%%%%%%%%%%%%%%%%%%%%%%%%%
%%%%% Solve function %%%%%
%%%%%%%%%%%%%%%%%%%%%%%%%%%%%%%%%%%
% Copyright (c) 2011-2015 by Thomas O'Hara, Yoram Rudy,
%                                        Washington University in St. Louis.
% All rights reserved.
%
% Redistribution and use in source and binary forms, with or without
% modification, are permitted provided that the following conditions are
% met:
%
% 1. Redistributions of source code must retain the above copyright notice,
% this list of conditions and the following disclaimer.
%
% 2. Redistributions in binary form must reproduce the above copyright
% notice, this list of conditions and the following disclaimer in the
% documentation and/or other materials provided with the distribution.
%
% 3. Neither the names of the copyright holders nor the names of its
% contributors may be used to endorse or promote products derived from
% this software without specific prior written permission.
%
% THIS SOFTWARE IS PROVIDED BY THE COPYRIGHT HOLDERS AND CONTRIBUTORS "AS
% IS" AND ANY EXPRESS OR IMPLIED WARRANTIES, INCLUDING, BUT NOT LIMITED
% TO, THE IMPLIED WARRANTIES OF MERCHANTABILITY AND FITNESS FOR A
% PARTICULAR PURPOSE ARE DISCLAIMED. IN NO EVENT SHALL THE COPYRIGHT
% HOLDERS OR CONTRIBUTORS BE LIABLE FOR ANY DIRECT, INDIRECT, INCIDENTAL,
% SPECIAL, EXEMPLARY, OR CONSEQUENTIAL DAMAGES (INCLUDING, BUT NOT
% LIMITED TO, PROCUREMENT OF SUBSTITUTE GOODS OR SERVICES; LOSS OF
% USE, DATA, OR PROFITS; OR BUSINESS INTERRUPTION) HOWEVER CAUSED AND
% ON ANY THEORY OF LIABILITY, WHETHER IN CONTRACT, STRICT LIABILITY,
% OR TORT (INCLUDING NEGLIGENCE OR OTHERWISE) ARISING IN ANY WAY OUT OF
% THE USE OF THIS SOFTWARE, EVEN IF ADVISED OF THE POSSIBILITY OF SUCH
% DAMAGE.

% MATLAB Implementation of the O'Hara-Rudy dynamic (ORd) model for the
% undiseased human ventricular action potential and calcium transient
%
% The ORd model is described in the article "Simulation of the Undiseased
% Human Cardiac Ventricular Action Potential: Model Formulation and
% Experimental Validation"
% by Thomas O'Hara, Laszlo Virag, Andras Varro, and Yoram Rudy
```

```
%
% The article and supplemental materails are freely available in the
% Open Access jounal PLoS Computational Biology
% Link to Article:
% http://www.ploscompbiol.org/article/info:doi/10.1371/journal.pcbi.1002061
%
% Email: tom.ohara@gmail.com / rudy@wustl.edu
% Web: http://rudylab.wustl.edu
% We use the function to solve currents and ion concentrations.

close all
clear all

%initial conditions for state variables
v=-87;
nai=7;
nass=nai;
ki=145;
kss=ki;
cai=1.0e-4;
cass=cai;
cansr=1.2;
cajsr=cansr;
m=0;
hf=1;
hs=1;
j=1;
hsp=1;
jp=1;
mL=0;
hL=1;
hLp=1;
a=0;
iF=1;
iS=1;
ap=0;
iFp=1;
iSp=1;
d=0;
ff=1;
fs=1;
fcaf=1;
fcas=1;
jca=1;
```

```
nca=0;
ffp=1;
fcafp=1;
xrf=0;
xrs=0;
xs1=0;
xs2=0;
xk1=1;
Jrelnp=0;
Jrelp=0;
CaMKt=0;

timeStep1=1;
Length_array=100000;
T_array1=zeros(Length_array,1);
T_array1(1) = 500;
options1 = odeset('MaxStep',0.1);             %     Set integrator options

%X0 is the vector for initial sconditions for state variables
X0=[v nai nass ki kss cai cass cansr cajsr m hf hs j hsp jp mL hL hLp a iF iS ap iFp iSp d ff fs
fcaf fcas jca nca ffp fcafp xrf xrs xs1 xs2 xk1 Jrelnp Jrelp CaMKt]';
CL=300;%pacing cycle length in ms
beats=10;%number of beats in the simulation
options=odeset('MaxStep',1);%options for ode solver
X = [];
T = [];

for m = [1:beats]
[TT XX]=ode23t(@model,[0 CL],X0',options1,1);
X = [X;XX];
T = [T;CL*(m-1)+TT];
X0 = XX(size(XX,1),:);
m
end


%rename values in the state variables vector
v=X(:,1);
nai=X(:,2);
nass=X(:,3);
ki=X(:,4);
kss=X(:,5);
cai=X(:,6);
cass=X(:,7);
```

```
cansr=X(:,8);
cajsr=X(:,9);
m=X(:,10);
hf=X(:,11);
hs=X(:,12);
j=X(:,13);
hsp=X(:,14);
jp=X(:,15);
mL=X(:,16);
hL=X(:,17);
hLp=X(:,18);
a=X(:,19);
iF=X(:,20);
iS=X(:,21);
ap=X(:,22);
iFp=X(:,23);
iSp=X(:,24);
d=X(:,25);
ff=X(:,26);
fs=X(:,27);
fcaf=X(:,28);
fcas=X(:,29);
jca=X(:,30);
nca=X(:,31);
ffp=X(:,32);
fcafp=X(:,33);
xrf=X(:,34);
xrs=X(:,35);
xs1=X(:,36);
xs2=X(:,37);
xk1=X(:,38);
Jrelnp=X(:,39);
Jrelp=X(:,40);
CaMKt=X(:,41);

% calculate and name dependent variables for the final beat in the
% simulation (i.e. currents and fluxes)
for i=[1:size(X,1)];
    IsJs=model(T(i),X(i,:),0);
    INa(i)=IsJs(1);
    INaL(i)=IsJs(2);
    Ito(i)=IsJs(3);
    ICaL(i)=IsJs(4);
    IKr(i)=IsJs(5);
```

```
        IKs(i)=IsJs(6);
        IK1(i)=IsJs(7);
        INaCa_i(i)=IsJs(8);
        INaCa_ss(i)=IsJs(9);
        INaK(i)=IsJs(10);
        IKb(i)=IsJs(11);
        INab(i)=IsJs(12);
        ICab(i)=IsJs(13);
        IpCa(i)=IsJs(14);
        Jdiff(i)=IsJs(15);
        JdiffNa(i)=IsJs(16);
        JdiffK(i)=IsJs(17);
        Jup(i)=IsJs(18);
        Jleak(i)=IsJs(19);
        Jtr(i)=IsJs(20);
        Jrel(i)=IsJs(21);
        CaMKa(i)=IsJs(22);
        Istim(i)=IsJs(23);
end

%cell geometry
L=0.01;
rad=0.0011;
vcell=1000*3.14*rad*rad*L;
vnsr=0.0552*vcell;
vjsr=0.0048*vcell;
Casr = (vjsr*X(:,8) + vnsr*X(:,9))/(vjsr+vnsr); %Ca2+ concentration in the SR

figure(1)
plot(T,v);
xlabel('T (ms)');
ylabel('V (mv)');
figure(2)
plot(T,X(:,6));
xlabel('T (ms)');
ylabel('Cai (mmol/L)');
figure(3)
plot(T,Casr);
xlabel('T (ms)');
ylabel('Casr (mmol/L)');
```

```
%figure1.m


%%%%%%%%%%%%%%%%%%%%%%%%%%%%%%%%%%%%
%%%%%figure1.m %%%%%%
%%%%%%%%%%%%%%%%%%%%%%%%%%%%%%%%%%%%
clear all;
close all;
clc;
load A11; %Calculated after 1000 beats in ischemic case 2 with CL=300ms; There are instructions about its content in the following
T3     =     A11(:,1);
V       =     A11(:,2);
Cai   =     A11(:,3);
Casr =     A11(:,4);
Jrel =     A11(:,5);
Ito   =     A11(:,6);
ICaL =     A11(:,7);



subplot(4,2,1)
plot(T3,V,'k','linewidth',1);
xlabel('T (ms)');
ylabel('V (mv)');
axis([0 3000 -100 50])

subplot(4,2,3)
plot(T3,Cai,'k','linewidth',1);
axis([0 3000 0 0.003])
xlabel('T (ms)');
ylabel('Ca_i (mmol/L)');

subplot(4,2,5)
plot(T3,Casr,'k','linewidth',1);
xlabel('T (ms)','FontSize',9);
ylabel('Ca_s_r (mmol/L)','FontSize',9);
axis([0 3000 2.5 4]);

a=find(T3==300);
b=find(T3==600);

subplot(4,2,7)
plot(T3(1:a(1)),Ito(1:a(1)),'k','linewidth',1);
```

```
hold on
plot(T3(a(2):b(1))-300,Ito(a(2):b(1)),'r--','linewidth',1);
xlabel('T (ms)');
ylabel('I_t_o (uA/uF)');
axis([-10 300 0 4]);



subplot(4,2,2)
plot(T3(1:a(1)),V(1:a(1)),'k','linewidth',1);
hold on
plot(T3(a(2):b(1))-300,V(a(2):b(1)),'r--','linewidth',1);
xlabel('T (ms)');
ylabel('V (mv)');
axis([-10 300 -100 50])
axes('position',[0.788714285714285 0.850938095238102 0.098 0.045],'FontSize',8);
plot(T3(12:48),V(12:48),'linewidth',1);
hold on
plot(T3(720:755)-300,V(720:755),'r--','linewidth',1);
axis([5 20 22 32]);

subplot(4,2,4)
plot(T3(1:a(1)),Cai(1:a(1)),'k','linewidth',1);
hold on
plot(T3(a(2):b(1))-300,Cai(a(2):b(1)),'r--','linewidth',1)
xlabel('T (ms)');
ylabel('Ca_i (mmol/L)');
axis([-10 300 0 0.003])
axes('position',[0.783357142857143 0.633981937602632 0.0980000000000001
0.045],'FontSize',8);
plot(T3(684:710),Cai(684:710),'k','linewidth',1);
hold on
plot(T3(1384:1411)-300,Cai(1384:1411),'r--','linewidth',1);
axis([290 300 0.00037 0.00041]);

subplot(4,2,6)
plot(T3(1:a(1)),Jrel(1:a(1)),'k','linewidth',1);
hold on
plot(T3(a(2):b(1))-300,Jrel(a(2):b(1)),'r--','linewidth',1)
xlabel('T (ms)');
ylabel('J_r_e_l (mM/ms)');
axis([-10 300 0 2]);
```

```
subplot(4,2,8)
plot(T3(1:a(1)),ICaL(1:a(1)),'k','linewidth',1);
hold on
plot(T3(a(2):b(1))-300,ICaL(a(2):b(1)),'r--','linewidth',1)
xlabel('T (ms)');
ylabel('I_C_a_L (uA/uF)');
axis([-10 300 -4 0]);


annotation('textbox',[0.0423 0.89 0.059 0.067],'String',{'a'},'LineStyle','none');
annotation('textbox',[0.0423 0.67 0.059 0.067],'String',{'b'},'LineStyle','none');
annotation('textbox',[0.0423 0.46 0.059 0.067],'String',{'c'},'LineStyle','none');
annotation('textbox',[0.0423 0.24 0.059 0.067],'String',{'d'},'LineStyle','none');
annotation('textbox',[0.475 0.89 0.059 0.067],'String',{'e'},'LineStyle','none');
annotation('textbox',[0.475 0.67 0.059 0.067],'String',{'f'},'LineStyle','none');
annotation('textbox',[0.475 0.46 0.059 0.067],'String',{'g'},'LineStyle','none');
annotation('textbox',[0.475 0.24 0.059 0.067],'String',{'h'},'LineStyle','none');
```

```
%figure2.m


%%%%%%%%%%%%%%%%%%%%%%%%%%%%%%%%%%%
%%%%%figure2.m %%%%%%
%%%%%%%%%%%%%%%%%%%%%%%%%%%%%%%%%%%
clear all;
close all;
clc;
subplot(2,1,1)
load A20; %Its contents are explained in the following
load A21; %Its contents are explained in the following
casr_case1a         = A20(:,1);    %Recordings of SR calcium ion concentration of each heart beat(the total number is 1000) in case 1 with CL = 250ms;
casr_case2a         = A20(:,2);    %Recordings of SR calcium ion concentration of each heart beat in case 2 with CL = 250ms;
casr_case3a         = A20(:,3);    %Recordings of SR calcium ion concentration of each heart beatin case 3 with CL = 250ms;
casr_cona           = A20(:,4);    %Recordings of SR calcium ion concentration of each heart beat in control condition with CL = 250ms;
carelease_case1a = A20(:,5);    %Recordings fo JSR released calcium ion concentration of each hear beat in case 1 with CL = 250ms;
carelease_case2a = A20(:,6);    %Recordings of JSR released calcium ion concentration    of each hear beat in case 2 with CL = 250ms;
carelease_case3a = A20(:,7);    %Recordings of JSR released calcium ion concentration of each hear beat in case 3 with CL = 250ms;
carelease_cona     = A20(:,8);    %Recordings of JSR released calcium ion concentration of each hear beat in control condition with CL = 250ms;

casr_case1b         = A21(:,1);    %Recordings of SR calcium ion concentration of each hear beat in case 1 with CL = 300ms;
casr_case2b         = A21(:,2);    %Recordings of SR calcium ion concentration of each hear beat in case 2 with CL = 300ms;
casr_case3b         = A21(:,3);    %Recordings of SR calcium ion concentration of each hear beat in case 3 with CL = 300ms;
casr_conb           = A21(:,4);    %Recordings of SR calcium ion concentration of each hear beat in control condition with CL = 300ms;
carelease_case1b = A21(:,5);    %Recordings of JSR released calcium ion concentration of each hear beat in case 1 with CL = 300ms;
carelease_case2b = A21(:,6);    %Recordings of JSR released calcium ion concentration of each hear beat in case 2 with CL = 300ms;
carelease_case3b = A21(:,7);    %Recordings of JSR released calcium ion concentration of each hear beat in case 3 with CL = 300ms;
carelease_conb     = A21(:,8);    %Recordings of JSR released calcium ion concentration of
```

each hear beat in control condition with CL = 300ms;

```
%cell geometry
L=0.01;
rad=0.0011;
vcell=1000*3.14*rad*rad*L;
Ageo=2*3.14*rad*rad+2*3.14*rad*L;
Acap=2*Ageo;
vmyo=0.68*vcell;
vnsr=0.0552*vcell;
vjsr=0.0048*vcell;
r_v = vjsr/(vnsr+vjsr);


plot(casr_case1a(91:638),carelease_case1a(91:638)./casr_case1a(91:638)*r_v,'r','linewidth',1);
hold on
plot(casr_case1a(639:1000),carelease_case1a(639:1000)./casr_case1a(639:1000)*r_v,'r.');
hold on

plot(casr_case2a(35:277),carelease_case2a(35:277)./casr_case2a(35:277)*r_v,'b','linewidth',1);
hold on
plot(casr_case2a(278:1000),carelease_case2a(278:1000)./casr_case2a(278:1000)*r_v,'bo');
hold on

plot(casr_case3a(19:166),carelease_case3a(19:166)./casr_case3a(19:166)*r_v,'m','linewidth',1);
hold on
plot(casr_case3a(167:1000),carelease_case3a(167:1000)./casr_case3a(167:1000)*r_v,'mx');
hold on

plot(casr_cona(8:128),carelease_cona(8:128)./casr_cona(8:128)*r_v,'g','linewidth',1);


xlabel('{SR Ca^2^+ content (mmol/L)}');
ylabel('{fractional SR Ca^2^+ release (%)}');

legend2 = legend('case 1','case 1','case 2','case 2','case 3','case 3','control');
set(legend2,'Position',[0.154 0.705 0.173 0.184]);

%axes('position',[0.2 0.6 0.15 0.3]);
axes('position',[0.79 0.62 0.08 0.12],'YTick',[0.106 0.112],'XTick',[2.7 2.8])
plot(casr_case1a(277:535),carelease_case1a(277:535)./casr_case1a(277:535)*r_v,'r','linewidth',
1);
hold on
plot(casr_case2a(102:185),carelease_case2a(102:185)./casr_case2a(102:185)*r_v,'b','linewidth'
```

```
,1);
hold on
plot(casr_case3a(54:90),carelease_case3a(54:90)./casr_case3a(54:90)*r_v,'m','linewidth',1);
hold on
plot(casr_cona(25:40),carelease_cona(25:40)./casr_cona(25:40)*r_v,'g','linewidth',1);
axis([2.7 2.8 0.106 0.112]);

annotation('textbox',[0.71 0.87 0.174 0.044],'String',{'CL = 250ms'},'FitBoxToText','off');

subplot(2,1,2)
plot(casr_case1b(97:1000),carelease_case1b(97:1000)./casr_case1b(97:1000)*r_v,'r','linewidth'
,1);
hold on
plot(casr_case2b(27:267),carelease_case2b(27:267)./casr_case2b(27:267)*r_v,'b','linewidth',1);
hold on
plot(casr_case2b(268:1000),carelease_case2b(268:1000)./casr_case2b(268:1000)*r_v,'b.');
hold on
plot(casr_case3b(14:156),carelease_case3b(14:156)./casr_case3b(14:156)*r_v,'m','linewidth',1);
hold on
plot(casr_case3b(157:1000),carelease_case3b(157:1000)./casr_case3b(157:1000)*r_v,'mx');
hold on
plot(casr_conb(5:95),carelease_conb(5:95)./casr_conb(5:95)*r_v,'g','linewidth',1);

xlabel('{SR Ca^2^+ content (mmol/L)}');
ylabel('{fractional SR Ca^2^+ release (%)}');

legend1 = legend('case 1','case 2','case 2','case 3','case 3','control');
set(legend1,'Position',[0.154 0.226 0.173 0.213]);
axes('position',[0.79 0.15 0.08 0.12],'YTick',[0.126 0.132],'XTick',[2.7 2.8]);
plot(casr_case1b(513:1000),carelease_case1b(513:1000)./casr_case1b(513:1000)*r_v,'r','linewi
dth',1);
hold on
plot(casr_case2b(137:268),carelease_case2b(137:268)./casr_case2b(137:268)*r_v,'b','linewidth
',1);
hold on
plot(casr_case3b(59:100),carelease_case3b(59:100)./casr_case3b(59:100),'m','linewidth',1);
hold on
plot(casr_conb(25:37),carelease_conb(25:37)./casr_conb(25:37)*r_v,'g','linewidth',1);

axis([2.7 2.8 0.126 0.132]);
annotation('textbox',[0.718 0.395 0.173 0.044],'String',{'CL = 300ms'},'FitBoxToText','off');
annotation('textbox',[0.023 0.93 0.059 0.044],'String',{'a'},'EdgeColor',[1 1 1]);
annotation('textbox',[0.023 0.465 0.059 0.044],'String',{'b'},'EdgeColor',[1 1 1]);
```

```
%figure3.m


%%%%%%%%%%%%%%%%%%%%%%%%%%%%%%%%%%%%
%%%%%figure3.m %%%%%%
%%%%%%%%%%%%%%%%%%%%%%%%%%%%%%%%%%%%
clear all;
close all;
clc;
subplot(2,1,1)
load A20; %Its contents are explained in the following
load A21; %Its contents are explained in the following
casr_case1a        = A20(:,1);   %Recordings of SR calcium ion concentration of each heart beat(the total number is 1000) in case 1 with CL = 250ms;
casr_case2a        = A20(:,2);   %Recordings of SR calcium ion concentration of each heart beat in case 2 with CL = 250ms;
casr_case3a        = A20(:,3);   %Recordings of SR calcium ion concentration of each heart beatin case 3 with CL = 250ms;
casr_cona          = A20(:,4);   %Recordings of SR calcium ion concentration of each heart beat in control condition with CL = 250ms;
carelease_case1a = A20(:,5);   %Recordings fo JSR released calcium ion concentration of each hear beat in case 1 with CL = 250ms;
carelease_case2a = A20(:,6);   %Recordings of JSR released calcium ion concentration  of each hear beat in case 2 with CL = 250ms;
carelease_case3a = A20(:,7);   %Recordings of JSR released calcium ion concentration of each hear beat in case 3 with CL = 250ms;
carelease_cona     = A20(:,8);   %Recordings of JSR released calcium ion concentration of each hear beat in control condition with CL = 250ms;

casr_case1b        = A21(:,1);   %Recordings of SR calcium ion concentration of each hear beat in case 1 with CL = 300ms;
casr_case2b        = A21(:,2);   %Recordings of SR calcium ion concentration of each hear beat in case 2 with CL = 300ms;
casr_case3b        = A21(:,3);   %Recordings of SR calcium ion concentration of each hear beat in case 3 with CL = 300ms;
casr_conb          = A21(:,4);   %Recordings of SR calcium ion concentration of each hear beat in control condition with CL = 300ms;
carelease_case1b = A21(:,5);   %Recordings of JSR released calcium ion concentration of each hear beat in case 1 with CL = 300ms;
carelease_case2b = A21(:,6);   %Recordings of JSR released calcium ion concentration of each hear beat in case 2 with CL = 300ms;
carelease_case3b = A21(:,7);   %Recordings of JSR released calcium ion concentration of each hear beat in case 3 with CL = 300ms;
carelease_conb     = A21(:,8);   %Recordings of JSR released calcium ion concentration of
```

each hear beat in control condition with CL = 300ms;

```
%cell geometry
L=0.01;
rad=0.0011;
vcell=1000*3.14*rad*rad*L;
Ageo=2*3.14*rad*rad+2*3.14*rad*L;
Acap=2*Ageo;
vmyo=0.68*vcell;
vnsr=0.0552*vcell;
vjsr=0.0048*vcell;
r_v = vjsr/(vnsr+vjsr);


plot(casr_case1a(91:638),carelease_case1a(91:638)./casr_case1a(91:638)*r_v,'r','linewidth',1);
hold on
plot(casr_case1a(639:1000),carelease_case1a(639:1000)./casr_case1a(639:1000)*r_v,'r.');
hold on

plot(casr_case2a(35:277),carelease_case2a(35:277)./casr_case2a(35:277)*r_v,'b','linewidth',1);
hold on
plot(casr_case2a(278:1000),carelease_case2a(278:1000)./casr_case2a(278:1000)*r_v,'bo');
hold on

plot(casr_case3a(19:166),carelease_case3a(19:166)./casr_case3a(19:166)*r_v,'m','linewidth',1);
hold on
plot(casr_case3a(167:1000),carelease_case3a(167:1000)./casr_case3a(167:1000)*r_v,'mx');
hold on

plot(casr_cona(8:128),carelease_cona(8:128)./casr_cona(8:128)*r_v,'g','linewidth',1);


xlabel('{SR Ca^2^+ content (mmol/L)}');
ylabel('{fractional SR Ca^2^+ release (%)}');

legend2 = legend('case 1','case 1','case 2','case 2','case 3','case 3','control');
set(legend2,'Position',[0.154 0.705 0.173 0.184]);

%axes('position',[0.2 0.6 0.15 0.3]);
axes('position',[0.79 0.62 0.08 0.12],'YTick',[0.106 0.112],'XTick',[2.7 2.8])
plot(casr_case1a(277:535),carelease_case1a(277:535)./casr_case1a(277:535)*r_v,'r','linewidth',
1);
hold on
plot(casr_case2a(102:185),carelease_case2a(102:185)./casr_case2a(102:185)*r_v,'b','linewidth'
```

```
,1);
hold on
plot(casr_case3a(54:90),carelease_case3a(54:90)./casr_case3a(54:90)*r_v,'m','linewidth',1);
hold on
plot(casr_cona(25:40),carelease_cona(25:40)./casr_cona(25:40)*r_v,'g','linewidth',1);
axis([2.7 2.8 0.106 0.112]);

annotation('textbox',[0.71 0.87 0.174 0.044],'String',{'CL = 250ms'},'FitBoxToText','off');

subplot(2,1,2)
plot(casr_case1b(97:1000),carelease_case1b(97:1000)./casr_case1b(97:1000)*r_v,'r','linewidth'
,1);
hold on
plot(casr_case2b(27:267),carelease_case2b(27:267)./casr_case2b(27:267)*r_v,'b','linewidth',1);
hold on
plot(casr_case2b(268:1000),carelease_case2b(268:1000)./casr_case2b(268:1000)*r_v,'b.');
hold on
plot(casr_case3b(14:156),carelease_case3b(14:156)./casr_case3b(14:156)*r_v,'m','linewidth',1);
hold on
plot(casr_case3b(157:1000),carelease_case3b(157:1000)./casr_case3b(157:1000)*r_v,'mx');
hold on
plot(casr_conb(5:95),carelease_conb(5:95)./casr_conb(5:95)*r_v,'g','linewidth',1);

xlabel('{SR Ca^2^+ content (mmol/L)}');
ylabel('{fractional SR Ca^2^+ release (%)}');

legend1 = legend('case 1','case 2','case 2','case 3','case 3','control');
set(legend1,'Position',[0.154 0.226 0.173 0.213]);
axes('position',[0.79 0.15 0.08 0.12],'YTick',[0.126 0.132],'XTick',[2.7 2.8]);
plot(casr_case1b(513:1000),carelease_case1b(513:1000)./casr_case1b(513:1000)*r_v,'r','linewi
dth',1);
hold on
plot(casr_case2b(137:268),carelease_case2b(137:268)./casr_case2b(137:268)*r_v,'b','linewidth
',1);
hold on
plot(casr_case3b(59:100),carelease_case3b(59:100)./casr_case3b(59:100),'m','linewidth',1);
hold on
plot(casr_conb(25:37),carelease_conb(25:37)./casr_conb(25:37)*r_v,'g','linewidth',1);

axis([2.7 2.8 0.126 0.132]);
annotation('textbox',[0.718 0.395 0.173 0.044],'String',{'CL = 300ms'},'FitBoxToText','off');
annotation('textbox',[0.023 0.93 0.059 0.044],'String',{'a'},'EdgeColor',[1 1 1]);
annotation('textbox',[0.023 0.465 0.059 0.044],'String',{'b'},'EdgeColor',[1 1 1]);
```

```
%figure4.m


%%%%%%%%%%%%%%%%%%%%%%%%%%%%%%%%%%%%
%%%%%figure4.m %%%%%%
%%%%%%%%%%%%%%%%%%%%%%%%%%%%%%%%%%%%
clear all;
close all;
clc;
load   A4; % Recordings of the values of Cansr, Cai and their ratios when alternans begin in different ischemic cases.
cansr = A4(:,1);
ph65 = 'Case 1';
ph66 = 'Case 2';
ph67 = 'Case 3';
case1a = repmat(ph65,4,1); %char. axis label
case2a = repmat(ph66,13,1);%char. axis label
case3a = repmat(ph67,8,1); %char. axis label
G = char(case1a,case2a,case3a);
subplot(2,2,1);

m=boxplot(cansr,G);
set(m,'LineWidth',1);
xlabel('Ischemic conditions');
ylabel('Ca_n_s_r (mmol/L)');


cai = A4(:,2);
ph65 = 'Case 1';
ph66 = 'Case 2';
ph67 = 'Case 3';
case1b = repmat(ph65,4,1);
case2b = repmat(ph66,13,1);
case3b = repmat(ph67,8,1);
G = char(case1b,case2b,case3b);
subplot(2,2,2);
m=boxplot(cai,G);
set(m,'LineWidth',1);
xlabel('Ischemic conditions');
ylabel('Ca_i (mmol/L)');

ratio = A4(:,3);
ph65 = 'Case 1';
ph66 = 'Case 2';
```

```
ph67 = 'Case 3';
case1c = repmat(ph65,4,1);
case2c = repmat(ph66,13,1);
case3c = repmat(ph67,8,1);
G = char(case1c,case2c,case3c);
subplot(2,2,3);
m=boxplot(ratio,G);
set(m,'LineWidth',1);
xlabel('Ischemic conditions');
ylabel('Ca_n_s_r/Ca_i');
```

```
%figure5.m

%%%%%%%%%%%%%%%%%%%%%%%%%%%%%%%%%%%%
%%%%%figure5.m %%%%%%
%%%%%%%%%%%%%%%%%%%%%%%%%%%%%%%%%%%%
clear all;
close all;
clc;

load A5; %There are instructions about its content in the following.
casrN = A5(:,1)./max(A5(:,1)); %normalized SR    calcium ion concentration
caiN    = A5(:,2)./max(A5(:,2)); %normalized cyctoplasmic calcium ion concentration
ratio = A5(:,3)./A5(:,2);           %A(:,3) represents the NSR calcium ion concentration. This value
shows the ratio of calcium ion concentration between NSR to the cytoplasm.

subplot(2,1,1)
plot(casrN,'.','Markersize',4);
hold on
plot(caiN,'.','Color',[0.85 0.16 0],'Markersize',4,'LineWidth',1);
xlabel('Number of Beats');
set(subplot(2,1,1),'LineWidth',1);

legend1 = legend('Normalized diastolic Ca_s_r','Normalized diastolic Ca_i');
set(legend1,'Position',[0.182 0.6 0.368 0.1],'LineWidth',1);

B1=300:330;
axes('position',[0.76 0.65 0.11 0.155]);
plot(B1,casrN(300:330),'.');
axis([300 330 0.82 0.84]);
set(axes,'LineWidth',1);

annotation('textbox',[0.145             0.85             0.162             0.0475],'String',{'CL             =
250ms'},'FitBoxToText','off','LineWidth',1);

subplot(2,1,2)
plot(ratio,'.','Markersize',4,'LineWidth',1);
xlabel('Number of Beats');
ylabel('Ca_n_s_r/Ca_i');
annotation('textbox',[0.145             0.37             0.162             0.0475],'String',{'CL             =
250ms'},'FitBoxToText','off','LineWidth',1);
set(subplot(2,1,2),'LineWidth',1);

annotation('textbox',[0.029 0.9 0.059 0.067],'String',{'a'},'LineStyle','none');
annotation('textbox',[0.029 0.466 0.059 0.067],'String',{'b'},'LineStyle','none');
```
